# Neuroimaging and molecular mechanism of action of Cang-ai volatile oil in the treatment of vascular cognitive impairment

**DOI:** 10.3389/fnins.2025.1688649

**Published:** 2025-11-03

**Authors:** Bojun Chen, Boshen Liang, Wei Li, Lei Wang, Jiaojian Wang, Lei Xiong, Fabao Gao

**Affiliations:** ^1^Yunnan University of Chinese Medicine, Kunming, China; ^2^Guangzhou University of Chinese Medicine, Guangzhou, China; ^3^Department of Radiology, West China Hospital of Sichuan University, Chengdu, China; ^4^Kunming University of Science and Technology, Kunming, China

**Keywords:** vascular cognitive impairment, Cang-ai volatile oil, neuroimaging, endoplasmic reticulum stress, inflammation, apoptosis

## Abstract

**Background:**

Cangai volatile oil (CAVO), as a traditional Chinese medicinal volatile oil, has been applied for its neuroprotective effects in conditions such as depression.

**Objective:**

To investigate whether CAVO can improve brain cognitive function in rats with vascular cognitive impairment using 7T high-field functional magnetic resonance imaging and molecular biology.

**Method:**

CAVO treatment was administered in a rat model of vascular dementia induced by bilateral permanent occlusion of the common carotid arteries (2 VO). The water maze was employed for behavioral assessment, while magnetic resonance imaging examined alterations in local homogeneity, low-frequency amplitude, and functional connectivity within the rat brain. Protein expression of inflammatory factors (NLRP3), apoptotic proteins (BAX, Bcl-2), and endoplasmic reticulum stress proteins (CHOP, PERK, GRP78) in the rat hippocampus was analyzed using Western blotting and PCR.

**Results:**

CAVO enhances functional connectivity strength in regions including the cingulate cortex-piriform cortex and anterior cingulate cortex-hypothalamus in VCI rats (*p* < 0.05). Simultaneously, CAVO reduced the expression of proteins in the endoplasmic reticulum stress pathway (CHOP, pPERK, CHOP, GRP78), the inflammatory factor NLRP3, and the apoptosis pathway (BAX, Bcl-2, Caspase3) (*p* < 0.05), increased Bcl-2 protein expression (*p* < 0.05). It also significantly reduced the mRNA expression of CHOP, NLRP3, GRP78, and BAX (*p* < 0.01).

**Conclusion:**

This study demonstrates that CAVO therapy reduces inflammatory responses in brain regions of VCI rats, decreases apoptosis and necrosis, protects neurons in affected areas, and simultaneously enhances functional connectivity strength between brain regions in VCI rats, thereby exerting a cognitive-improving effect on VCI.

## 1 Introduction

Vascular cognitive impairment (VCI) is a form of cognitive dysfunction caused by various cerebrovascular diseases and their associated risk factors. In China, VCI accounts for 1.6% of dementia cases among people over 60 years of age, where the overall prevalence is 6%. The risk of VCI doubles approximately every 5.3 years, making it the second leading cause of dementia after Alzheimer’s disease. Epidemiologic findings suggest that 81 million people may be living with dementia in developing countries worldwide by 2040, with VCI accounting for about 30% of these cases. The rising incidence and prevalence of VCI globally, driven by general population aging, is a growing concern (O’Brien and Thomas, 2015; Morgan and Mc Auley, 2024). Research indicates that ischemic stroke, hemorrhagic stroke, and cerebral ischemia-hypoxia can all lead to vascular dementia. Age and major traditional vascular risk factors (smoking, diabetes, and hypercholesterolemia) account for the majority of cardiovascular and cerebrovascular event risks, making such individuals susceptible to vascular dementia (Etherton-Beer, 2014). Current treatments for VCI primarily include cholinesterase inhibitors (e.g., donepezil, galantamine, and carboplatin) and noncompetitive N-methyl-D-aspartate receptor antagonists (memantine) (Chinese Stroke Association Vascular Cognitive Impairment Subcommittee, 2024); however, these therapeutic agents increase the risk of adverse effects, such as malignancy and diarrhea.

Herbal compounding and traditional Chinese medicine offer several therapeutic advantages, but their underlying mechanisms remain unclear. Cang-ai volatile oil (CAVO) is a herbal compound preparation composed of Cangzhu, Ai Ye, Huo Xiang, Pei Lan, Clove, and other ingredients. Preliminary studies have found that CAVO has great anti-inflammatory effects (Zhang et al., 2021). Inflammatory factors are promoters that directly activate endoplasmic reticulum stress (ERS) (Xu et al., 2021), thereby affecting cell survival and apoptosis (Hu et al., 2019). The inhibition of ERS in the brains of rats with VCI has been shown to effectively improve cognitive deficits (Tanaka and Kawahara, 2017). Preliminary studies indicate that CAVO can improve depressive-like behaviors and cognitive function (Chen et al., 2019). Based on this, we hypothesized that CAVO may enhance cognitive function in VCI rats by regulating ERS through its modulation of inflammatory factors.

Neuroimaging is a key method for VCI risk prediction, etiologic–pathologic diagnosis, and prognostic assessment. Functional magnetic resonance imaging (fMRI) is increasingly used to reliably assess the relationship between neural activity and whole-brain processing. Resting-state functional magnetic resonance (rs-fMRI) and related analytical techniques have been applied to ischemic cerebrovascular disease. These methods enable the observation of oxygenation and deoxyhemoglobin levels [through blood oxygen level-dependent (BOLD) signals] in the brain in a near-physiological state, reflecting neuronal activity. Regional homogeneity (ReHo) is defined as cluster time-dependent synchronization (Zhu et al., 2022) and can indirectly assess neural markers of localized brain activity. Previous studies have demonstrated reduced overall functional connectivity (FC) strength in the brains of patients with cognitive decline (Wang et al., 2019).

To test our hypothesis, we created a VCI rat model using bilateral permanent common carotid artery ligation, assessing the model’s success by evaluating the rats’ behaviors. We used rs-fMRI to evaluate changes in local coherence (ReHo) values and FC strength in brain regions of the VCI rats before and after CAVO treatment. Additionally, we used pathological staining, Western blot, polymerase chain reaction (PCR), immunofluorescence, and transmission electron microscopy to investigate whether CAVO played a neuroprotective role in the ERS/NLRP3/BAX pathway and explore the effects of CAVO on the cognitive function of the VCI brain.

## 2 Materials and methods

### 2.1 Animal model established

Forty-eight male Sprague-Dawley rats weighing 200 ± 20 g were purchased from Chengdu Dashuo Laboratory Animal Co. The rats were divided into Sham-operated (*n* = 12) and experimental (*n* = 36) groups. They were domesticated and fed for 1 week before the experiment, housed at a temperature of 21.0 °C ± 1.0 °C and 50% humidity, under a standard light/dark cycle. They were fed for 3 days, then feeding was ceased for 12 h to establish the model. Bilateral permanent common carotid artery ligation was used to establish the VCI animal model (unilateral two-times modeling). After being fasted for 12 h, the rats were anesthetized with 1% sodium pentobarbital (40 mg/kg, intraperitoneal) to ensure spontaneous breathing during the procedure. Rats were secured in the supine position with the head fixed. After hair removal and skin preparation, the neck was sterilized, and an incision was made along its middle, separating the bilateral common carotid arteries. The wound was closed using a No.0 mousse ligature suture. The 12 rats in the Sham-operated group underwent a similar procedure, only without ligation of the common carotid arteries—the skin was cut and separated from the tissues before suturing (Pang et al., 2020). The rats in both groups were injected with penicillin 0.2 U/d for three consecutive days to prevent infections, before being returned to the laboratory for further rearing once their activity had returned to normal (Xiuqin et al., 2024). After 3 days of penicillin injection, the Morris water maze was performed to test their spatial learning and memory. The escape latency of the rats in the Sham-operated group was taken as the reference value, and the ratio of the escape latency to the reference value was calculated for the rats in the remaining groups. If the ratio was greater than 20%, the rats were deemed cognitively impaired, indicating that the model was successfully established. The rats in the experimental group with confirmed cognitive impairment were then randomly divided into model, donepezil, and CAVO treatment groups.

### 2.2 CAVO preparation

CAVO comprises multiple Chinese herbal ingredients. All raw materials were procured from Yunnan Hehe Chinese Herbal Medicine Slices Co., Ltd. and extracted using steam distillation. The herbs were weighed proportionally, pulverized into powder, mixed, and steeped in eight times their volume of water for 4 h before extraction for 6 h. The total essential oil yield was 3.6% (v/w). The extract was dried over anhydrous sodium sulfate and stored in brown glass containers at 4 °C (Zhou et al., 2024).

### 2.3 Experimental groupings

1. Cang-ai treatment group (CAVO): 1.5% → 0.9 mL of Cang-ai oil dissolved in 60 mL of saline. CAVO was administered by gavage, at a dose of approximately 1/30 of LD50 (3.86 mL-kg-1-d-1), i.e., 0.12 mL-kg-1-d-1 for 14 days (Huang et al., 2023).
2. Donepezil group (Donepezil): Donepezil was administered by gavage at a dose of 0.5 mg/(kg-d), and the gavage concentration was standardized at 1 mL/100 g of rat body weight daily for 14 days.
3. Model group (VCI): normal feeding, gavage of equal doses of saline for 14 days.
4. Sham operation group (Sham): normal feeding, gavage with equal doses of saline for 14 days.

### 2.4 Morris water maze

The experimental setup for the water maze test comprised a circular pool. The pool was equipped with an automatic camera fixed on top (100 cm in diameter and 50 cm above the ground). The pool was divided into quadrants—I, II, III, and IV. A platform was added to Quadrant I, in the form of a circular plane with a diameter of 5 cm, placed 1 cm below the level of the pool. Before the experiment, ink was injected into the water to enhance the contrast of the experimental images. The water temperature was maintained at 22 °C–24 °C. The experiment was divided into two phases: a directional navigation experiment and a space exploration experiment. Before the experiment, the pool was filled with water to a depth of 1–2 cm above the platform. During the acquisition phase (Days 1–4), mice were trained once daily and placed in each of the four quadrants for 1 min of platform search. Mice failing to locate the platform within 1 min were guided by the experimenter to remain on the platform for 30 s. During the test phase (Day 5), the platform was removed, and a 60-second exploration test was conducted to assess the mice’s memory of the platform location (Zheng et al., 2022).

### 2.5 Rs-fMRI scanning

MRI scans were performed on rats over 3 days. Animals were anesthetized with 3% isoflurane mixed with an oxygen flow rate of 1 L/min, fixed in the prone position in a head coil. Pressure sensors were placed in the submental area of the strongest respiration for respiratory gating. Four electrodes of an MRI-specific cardiac monitor were inserted into the rats’ limbs for electrocardiogram (ECG) monitoring. MRI scanning was performed after the ECG waveform and respiratory rate had stabilized. A heating blanket was placed under the abdomen of the rats to maintain their body temperature.

rs-fMRI was performed using a 2DT2* weighted single excitation gradient echo planar imaging (EPI) sequence (2D GEEPI; TR 2,000 ms; TE 29 ms; 20 0.7 mm coronal slices with 0.1 mm slice gaps; 300 repetitions, frequency coded left–right). The field of view (FOV) was (30 × 30) mm^2^ with a matrix size of (128 × 128), resulting in voxel dimensions of (0.234 × 0.234 × 0.8) mm^3^. rs-fMRI scans were acquired 40 min after the initial doughnut injection of the administered drug, and the total scanning duration was 10 min. The slicing packet covered a large portion of the brain, ranging from approximately 5.16 mm fontanelle to bregma −8.88 mm.

Diffusion tensor imaging scans were acquired using a dual-excitation spin-echo (SE) EPI sequence (2D DW-SE-EPI; TR7500ms; TE26ms; 20 0.7 mm coronal slices with a slice gap of 0.1 mm, *b* value of 800 s/mm^2^, diffusion gradient pulse; duration Δ4ms, diffusion gradient separation Δ12ms, frequency coded left–right). Fifteen b0 images were acquired (*B* = 0 s/mm^2^; 5 b0 images per 20 diffusion-weighted images), with a total acquisition time of approximately 20 min. The FOV was (30 × 30) mm^2^, the acquisition matrix was (128 × 128), and the resolution was (0.234 × 0.234 × 0.8) mm^3^. The slice package was similar to that of the rs-fMRI acquisition.

3D images were acquired using a 3D RARE sequence (TR3185ms; TE44ms; RARE factor 8). The matrix size was (256 × 64 × 50), the FOV was (29 × 16 × 10.2) mm^3^, the voxel resolution was (0.11 × 0.25 × 0.20) mm^3^, and the total scan duration was 43 min.

### 2.6 Rs-fMRI analysis

#### 2.6.1 Data preprocessing

Spikes were first removed (motion scrubbing), and the mean functional image was calculated. The T2w and mean functional images were then skull-stripped. The FLIRT function in the FSL toolkit was used to align the skull-removed T2w image to the averaged functional image, and the aligned-to-functional T2w image was aligned to the standard template to generate a bold reference. Based on the bold reference, the functional image was aligned to the standard template. The standard template uses SIGMA, which enables linear and nonlinear alignment of multimodal acquisitions to accurately identify brain regions involved in physiological or pathophysiological states. SPM’s built-in functions were then used to correct for motion. The ANTS toolkit was used to nonlinearly (deformable) align fMRI images to structural templates to correct for distortions.^1^ Independent component analysis cleaning was run on a single scan (IC = 50) to manually identify and mark bad components. Regression was then performed on the bad IC components, motion parameters, white matter, and cerebrospinal fluid (CSF) signals. After regression, spatial smoothing and temporal filtering were conducted, using 1 mm Gaussian kernel smoothing with 0.01–0.1 bandpass filtering (Barrière et al., 2019).

#### 2.6.2 Analysis of rat rs-fMRI data

ReHo metrics were calculated at the voxel level using DPABI’s built-in functions. Statistical analyses were performed using DPABI, including multivariate analysis of variance (ANOVA), setting a significance threshold of *p* < 0.01 and screening for significant clusters (clusters) with voxel counts greater than 20. In addition, *post hoc* comparisons were performed using a two-sample *t*-test (two-sample *t*-test).

FC analysis was based on SIGMA mapping [containing 59 regions of interest (ROIs)]. First, mean time series were extracted from each ROI, and the Pearson correlation coefficients were calculated between ROIs to construct a 59 × 59 FC matrix for each subject. Subsequently, gradient analysis was performed using the BrainSpace toolkit with the normalized angle kernel function and dimensionality reduction using principal component analysis. To ensure comparability of gradients across individuals, Procrustes alignment was applied (Vos De Wael et al., 2020).

### 2.7 Pathologic staining and immunofluorescence

#### 2.7.1 HE staining

Following the MRI scanning, the brain tissues of all animals were collected for histologic analysis. Rats were anesthetized with excess isoflurane before being perfused with 250 mL of ice saline, followed by 300 mL of 4% paraformaldehyde (PFA) at a rate of 12 mL/min in 0.1 mol/L phosphate buffer solution for 30 min. After perfusion, brain tissues were removed and fixed in 40 mL of 4% paraformaldehyde for adequate fixation. Subsequently, the brain tissue was embedded in paraffin. Coronal sections, 4 μm thick, were then obtained serially from approximately the posterior cerebral region (−2.36 mm) using a sled slicer.

#### 2.7.2 Nissl staining

The obtained tissue sections were mounted on slides, deparaffinized, and stained with a 1% cresyl violet solution using Nissl staining for hippocampal histomorphometry. Then, the cellular structures within the Nissl vesicle neurons in the CA1 brain region of the hippocampus were visualized under a light microscope (CM3000; Bensheim Leica).

#### 2.7.3 Immunofluorescence

Brain tissues were paraffin-embedded and cut into 4 μm sections. Paraffin sections were dewaxed with xylene, followed by dehydration with a gradient of ethanol solutions. After rehydration, sections were washed with distilled water. Sections were then placed in buffer solution. Allow to cool naturally. Decolorize with PBS (pH 7.4) and wash three times on a shaking platform (5 min each). Incubate with 3% hydrogen peroxide solution for 15 min at room temperature in the dark. Wash three times with PBS (5 min each). Add 5% goat serum (or rabbit serum if the primary antibody is goat-derived) for 30 min to block. Discard the blocking solution. Add the primary antibody diluted in PBS [CD31 (Cat. No. GB11063-2), BAX (Cat. No. GB11690) diluted 1:200, CHOP (Cat. No. 15204-1-AP) diluted 1:400] and incubate overnight at 4 °C. The next day, wash three times with PBS (5 min each). Add species-specific goat anti-rabbit IgG secondary antibody (Catalog No. GAR0072) and incubate at 37 °C in the dark for 40 min. Prepare fresh TSA working solution containing 0.03% H₂O₂ by diluting TSA buffer. Add 100 μL TSA solution to each slide and incubate at room temperature in the dark for 10 min. Wash three times with PBS (5 min each). Repeat the TSA staining steps after antigen retrieval (excluding the primary antibody step). Finally, add DAPI staining. The methodology used was adapted from previous research (Wang et al., 2022).

#### 2.7.4 Transmission electron microscopy

The hippocampal tissue was sectioned into 1 mm^3^ plaques and placed in 2.5% glutaraldehyde for 2 ~ 4 h at 4 °C for cryopreservation to observe the ultrastructural changes to hippocampal cells. Small specimens of brain tissue were taken, fixed with 1% osmium tetroxide, dehydrated with graded ethanol, further cut into ultrathin sections, stained with uranyl acetate and lead citrate, and finally observed and photographed by transmission electron microscopy (TEM) (Chen et al., 2024).

### 2.8 Elisa

An equal volume of RIPA buffer containing protease inhibitors was added to the hippocampal tissue. The mixture was then dissolved in ice for 30 min with gentle stirring. Fragments were subsequently removed by centrifugation. Aliquots of lysis products were removed according to the manufacturer’s instructions and assayed using an enzyme-linked immunosorbent assay (ELISA) kit (Ma et al., 2025). TNF-α, Elabscience, E-EL-R2856; IL-1β, Elabscience, E-EL-R0012; IL-6, Elabscience, E-EL-R0015; IL-18, Elabscience, E-EL-R0567.

### 2.9 Western blot

Proteins were extracted from rat hippocampal tissue using RIPA lysis buffer, followed by centrifugation, shaking, and homogenization. Nuclear proteins were extracted using a nuclear extraction kit in accordance with the manufacturer’s protocol. Protein concentration was determined using the bicinchoninic acid assay (BCA) kit (BL521A, Biosharp, Beijing, China), and 10% ~ 12% sodium dodecyl sulfate-polyacrylamide gel electrophoresis (SDS-PAGE) was used to separate total and nuclear proteins from hippocampal tissues. These tissues were then transferred onto PVCIF membranes by wet transfer. The membrane was closed with 5% skimmed milk solution on a 4 °C shaker for 2 h. The primary antibody was incubated overnight, and the secondary antibody for 1 h. Band intensities were analyzed using ImageJ software (Peng et al., 2022). *β*-actin was used as a loading control protein. Primary antibody information: *β*-Actin, Cat No. AF7018 1:5,000; CHOP, Cat No. AF6277, 1:1,000; PERK, Proteintech, Cat No. 10701-1-AP, 1:1,000; p-PERK, Cat No. AF5304 1:1,000; caspase-3, Cat No. AF6311; GRP78, Cat No. AF5366; NLRP3, Cat No. DF15549, 1:1,000. Secondary antibody information: goat anti-rabbit IgG (H + L) HRP, MULTI SCIENCES, Cat No. 1 GAR0072; goat anti-mouse IgG (H + L) peroxigen peroxidase, MULTI SCIENCES, Cat No. GAM0072.

### 2.10 PCR

Total RNA was extracted from the cerebral cortex and hippocampus of the rats using the Universal RNA Extraction Kit. Total RNA to cDNA RT was analyzed using PrimeScript RT Master Mix. Real-time PCR was performed using a fluorescent thermocycler iQ5 Thermocycler (Bio-Rad) with TB Green Premix Ex Taq II according to the manufacturer’s instructions. mRNA levels were detected using the 2^−ΔΔCT^ mathematical model (Shen et al., 2021). Primer information is shown in Table 1.

**Table 1:** mRNA primer information.

Gene	Species	Direction	Primer sequence (5′-3′)
*β*-Actin	Rat	Forward	TGTCACCAACTGGGACGATA
		Reverse	GGGGTGTTGAAGGTCTCAAA
PERK	Rat	Forward	TGGGATGTCGCCGATGGGATAG
		Reverse	AATTCCACTTCTCACTGCCGCTTC
NLRP3	Rat	Forward	GCCGTCTACGTCTTCTTCCTTTCC
		Reverse	CATCCGCAGCCAGTGAACAGAG
BAX	Rat	Forward	GCGAGTGTCTCAGGCGAATTGG
		Reverse	AGTCTGTATCCACATCAGCAATCATCC
CHOP	Rat	Forward	TACTCTTGACCCTGCATCCCTAGC
		Reverse	TCCTCCTGAGCCATAGAACTCTGAC
Bcl-2	Rat	Forward	TGGAGAGCGTCAACAGGGAGATG
		Reverse	GGTGTGCAGATGCCGGTTCAG

PERK, stay endoplasmic reticulum kinase; CHOP, C/EBP homologous protein; NLRP3, inflammatory factor NOD-like receptor heat protein structural domain-associated protein 3; Bcl-2, B lymphoblastoma-2 gene; BAX, Bcl-2-associated X protein.

### 2.11 Statistical analysis

GraphPad Prism version 9.5.0 (GraphPad Software, San Diego, CA, USA) was used to perform statistical analysis and produce graphs. Measurement data were analyzed by one-way ANOVA to determine the overall significant differences, and the LSD method was used to compare the two groups if the variance was uniform. If the variance was not uniform, the rank sum test was used. Imaging statistics were also analyzed by multifactorial ANOVA, and *post hoc* comparisons were conducted using a two-sample *t*-test, with *p* < 0.05 indicating that the differences were statistically significant.

## 3 Results

### 3.1 Morris water maze test

During the experiment, two rats perished due to surgical complications. On day 1 of training, the escape latency of rats in the VCI and CAVO groups was elevated compared to that of the Sham group (*p* < 0.05). No significant differences were observed in the escape latency of rats in the CAVO group on day 1 of the experiment compared to the VCI group, and there was a decrease in the escape latency of rats in the Donepezil group on day 1 of the experiment compared to the VCI group.

The VCI group’s escape latency was significantly higher on day 4 of training than that of the Sham group (*p* < 0.01). On day 4 of the experiment, the CAVO group’s escape latency was significantly lower than that of the VCI group (*p* < 0.01), and the Donepezil group’s escape latency was lower than that of the VCI group (*p* < 0.01).

**Figure 1:**
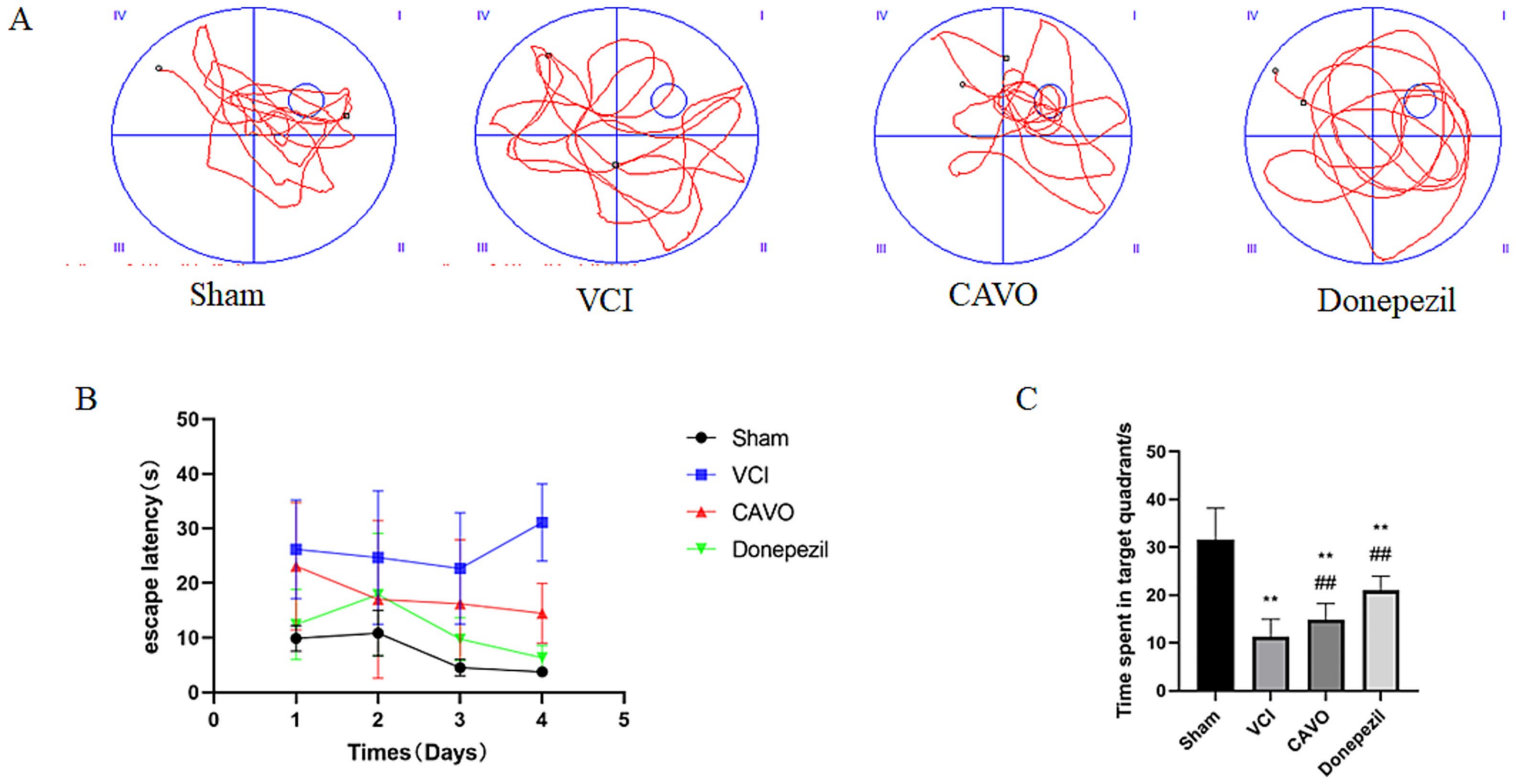
Morris water maze test results. **(A)** Morris water maze movement trajectories of rats in the Sham, VCI, CAVO, and Donepezil groups after treatment. **(B)** Time to escape for rats in each group during the 4-day training period. **(C)** Dwell time in the target quadrant after the platform was withdrawn in each group of rats. Compared with the Sham group, *p < 0.05, **p < 0.01; compared with the VCI group, #*p* < 0.05, ##*p* < 0.01. * Indicates statistical difference from the Sham group and # indicates statistical difference from the VCI group.

In addition, the residence time in the target quadrant was significantly lower (*p* < 0.05) in the VCI group than in the Sham group, significantly elevated (*p* < 0.05) in the CAVO group compared to the VCI group, and significantly higher (*p* < 0.01) in the Donepezil group compared to the VCI group (see Figure 1 and Table 2).

**Table 2:** Escape latency time of rats in each group (x ± s, *n* = 6).

Group	Sham	VCI	CAVO	Donepezil
Escape latency period—Day 1	9.91 ± 2.36	26.20 ± 9.04^*^	23.12 ± 11.70^*^	12.42 ± 6.44^#^
Escape latency period—Day 2	10.86 ± 4.17	24.67 ± 12.24	17.03 ± 14.43	17.88 ± 11.23
Escape latency period—Day 3	4.52 ± 1.48	22.71 ± 10.20^**^	16.23 ± 11.73	9.77 ± 3.92
Escape latency period—Day 4	3.76 ± 0.83	31.11 ± 7.03^**^	14.48 ± 5.48^**##^	6.34 ± 2.28^##^
Target quadrant stay time	31.60 ± 6.55	11.21 ± 3.78^**^	14.83 ± 3.36^**#^	21.01 ± 2.93^**##^

*p < 0.05, **p < 0.01 compared to Sham group; #*p* < 0.05, ##*p* < 0.01 compared to VCI group.

### 3.2 Imaging results

#### 3.2.1 ReHo results

**Figure 2:**
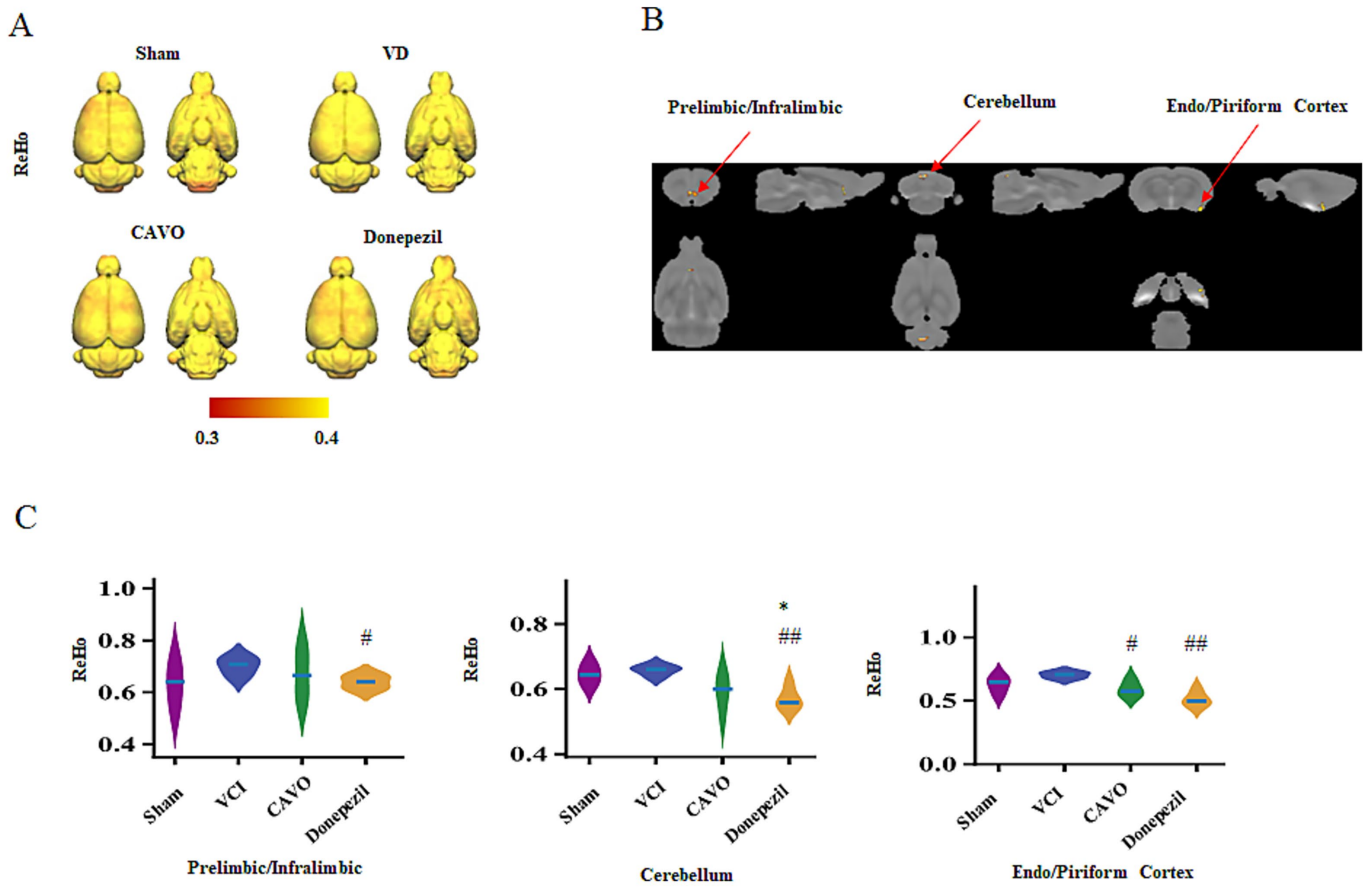
Results of ReHo analysis. **(A)** Mean ReHo values in the brains of rats in the Sham, VCI, CAVO, and Donepezil groups were quantified using the SIGMA standard template. **(B)** Functional maps of the differential brain regions [limbic cortex, pyriform cortex (piriform cortex), endopiriform cortex (endopiriform cortex), and cerebellum (cerebellum)] resulting from ANOVA. **(C)** ReHo values in each differential brain region [limbic cortex, pyriform cortex (piriform cortex), and cerebellum (cerebellum)] of rats in each group. Compared with the Sham group, *p < 0.05; compared with the VCI group, #*p* < 0.05 and ##*p* < 0.01.

ReHo analysis screened out three groups of differential brain areas. The VCI group had higher ReHo values in the limbic cortex (prelimbic/infralimbic) than the Sham group, and the CAVO and Donepezil groups had lower ReHo values compared with the VCI group (*p* < 0.05). In the endopiriform cortex, the VCI group had higher ReHo values than the Sham group, and the CAVO group had lower ReHo values than the VCI group (*p* < 0.05). In the piriform cortex, ReHo values were increased in the VCI group compared to the Sham group and decreased in the CAVO and Donepezil groups compared to the VCI group (*p* < 0.05). In the cerebellum, ReHo values were increased in the VCI group compared to the Sham group and decreased in the CAVO and Donepezil groups compared to the VCI group (*p* < 0.05). Overall, the Donepezil group had decreased ReHo values (*p* < 0.01) (see Figure 2 and Table 3).

**Table 3:** Comparison of ReHo values among groups of rats (x ± s, *n* = 6).

Group	Sham	VCI	CAVO	Donepezil
Prelimbic/infralimbic	0.62 ± 0.05	0.70 ± 0.02	0.59 ± 0.05^#^	0.51 ± 0.05^##^
Cerebellum	0.64 ± 0.02	0.65 ± 0.01	0.59 ± 0.05	0.56 ± 0.03^*##^
Endo/piriform cortex	0.63 ± 0.07	0.70 ± 0.03	0.67 ± 0.08	0.63 ± 0.02^#^

*p < 0.05 compared to Sham group; #*p* < 0.05 and ##*p* < 0.01 compared to VCI group.

#### 3.2.2 Gradient imaging results

**Figure 3:**
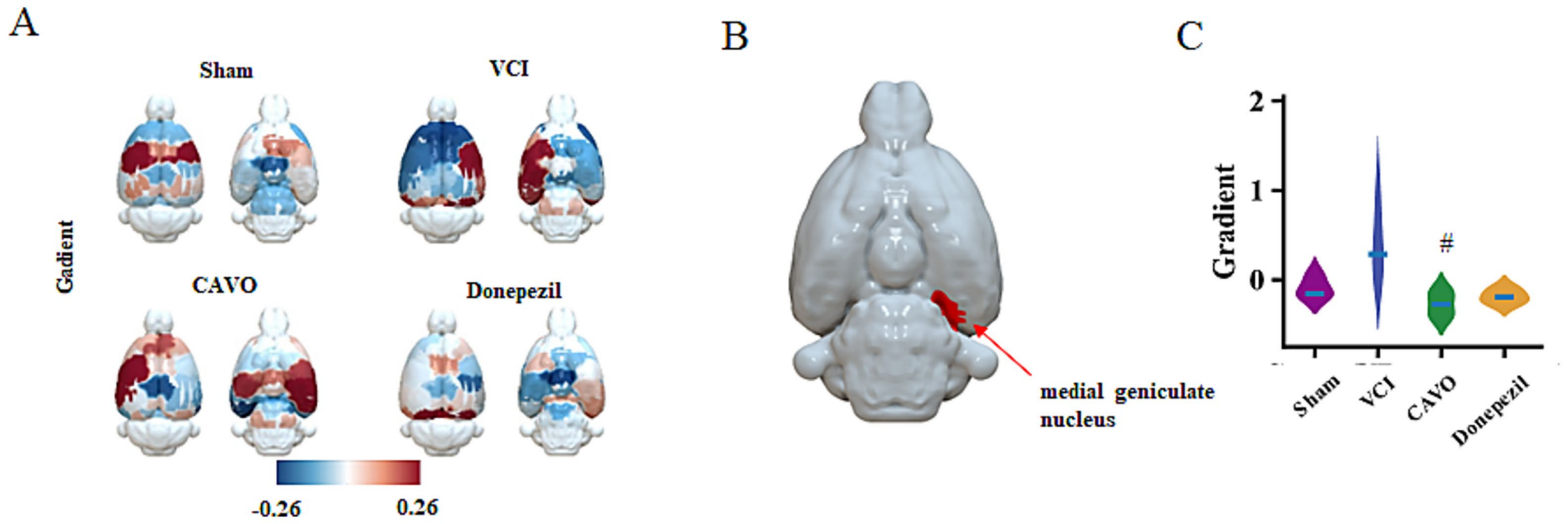
Gradient imaging results. **(A)** Quantification of the average gradient values in the brains of rats in Sham, VCI, CAVO, and Donepezil groups using the SIGMA standard template. **(B)** Differential brain regions [medial geniculate nucleus (MGN)] obtained after ANOVA. **(C)** Statistics of gradient values in the brain region of the medial geniculate nucleus (MGN) in each group of rats. #*p* < 0.05 compared with the VCI group.

Gradient analysis screened a total of one group of differential brain regions. In the medial geniculate nucleus (MGN), the gradient value was increased in the VCI group compared with the Sham group, the gradient value was decreased in the CAVO group compared with the VCI group (*p* < 0.05), and there was no statistically significant difference between the Donepezil and VCI groups (see Figure 3 and Table 4).

**Table 4:** Comparison of gradient values in MGN of rats in each group (x ± s, *n* = 6).

Group	Sham	VCI	CAVO	Donepezil
Gradient-medial geniculate nucleus	−0.09 ± 0.10	0.40 ± 0.36	−0.27 ± 0.12^#^	−0.18 ± 0.07

#*p* < 0.05 compared to VCI group.

#### 3.2.3 Results of functional connectivity analysis

FC was screened in a total of five groups to examine specific brain region connectivity. In the cingulate cortex–piriform cortex, FC strength was reduced in the VCI group compared with the Sham group, was elevated in the CAVO group compared with the VCI group (*p* < 0.05), and no statistically significant difference was observed between the Donepezil and the VCI groups.

FC strength in the prelimbic cortex-hypothalamus was reduced in the VCI group compared to the Sham group, and was elevated in the CAVO and Donepezil groups compared to the VCI group (*p* < 0.05).

FC strength in the primary somatosensory–subcoeruleum/pontine reticular nucleus was reduced in the VCI group compared to the Sham group, and increased in the CAVO and Donepezil groups compared to the VCI group (*p* < 0.05).

FC strength in the dorsal hippocampus-coliculus was increased in the VCI group compared to the Sham group. FC strength was decreased in the CAVO and Donepezil groups compared to the VCI group (*p* < 0.05).

**Figure 4:**
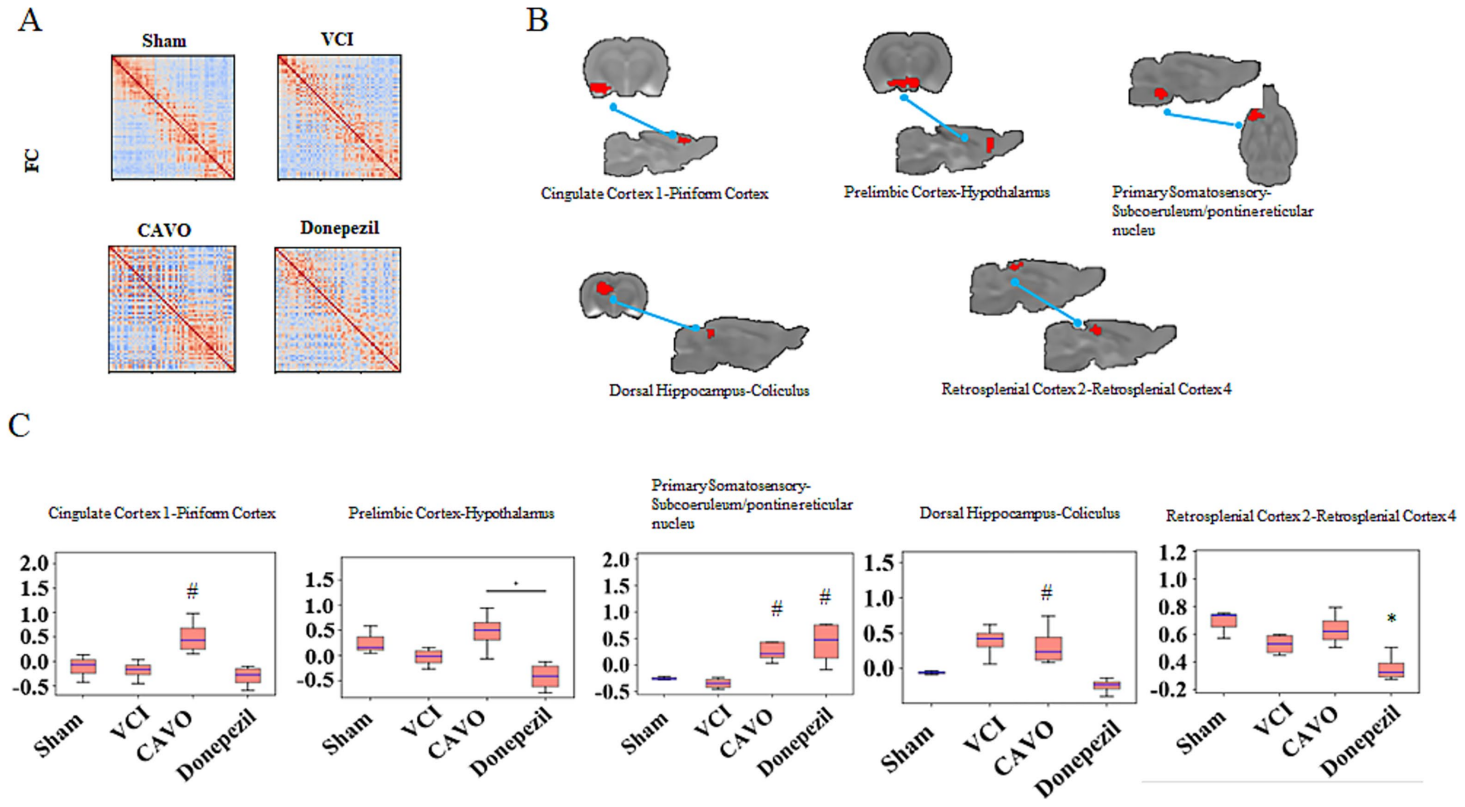
Results of functional connectivity analysis of rat brains. **(A)** Quantification of the average gradient values in the brains of rats in the Sham, VCI, CAVO, and Donepezil groups using the SIGMA standard template. **(B)** Functional maps of differential brain regions obtained after ANOVA. **(C)** Statistics of gradient values in each differential brain region for each group of rats. *p* < 0.05 for the CAVO and Donepezil groups* compared with the Sham group; *p* < 0.05 for the CAVO and Donepezil groups# compared with the VCI group.

FC strength in the retrosplenial cortex 2–retrosplenial cortex 4 was lower in the VCI group than in the Sham group, higher in the CAVO group than in the VCI group, and higher in the Donepezil group than in the VCI group (see Figure 4 and Table 5).

**Table 5:** Comparison of the strength of functional connectivity among groups of rats (x ± s, *n* = 6).

Group	Sham	VCI	CAVO	Donepezil
Cingulate cortex 1-piriform cortex	−0.12 ± 0.20	−0.17 ± 0.15	0.48 ± 0.33^#^	−0.32 ± 0.20
Prelimbic cortex-hypothalamus	0.26 ± 0.20	−0.03 ± 0.14	0.43 ± 0.37	−0.38 ± 0.26
Primary somatosensory-subcoeruleum/pontine reticular nucleus	−0.22 ± 0.03	−0.32 ± 0.08	0.37 ± 0.35^#^	0.41 ± 0.38^#^
Dorsal hippocampus-coliculus	−0.06 ± 0.01	0.36 ± 0.20	0.28 ± 0.25	−0.23 ± 0.09^*#^
Retrosplenial cortex 2–retrosplenial cortex 4	0.61 ± 0.08	0.43 ± 0.06	0.58 ± 0.14	0.24 ± 0.09^*#^

*p < 0.05, compared with Sham group; #*p* < 0.05, compared with VCI group.

### 3.3 Effects of CAVO on ERS pathway in VCI rats

#### 3.3.1 Expression of serum inflammatory factors in VCI rats treated with CAVO detected by ELISA

**Figure 5:**
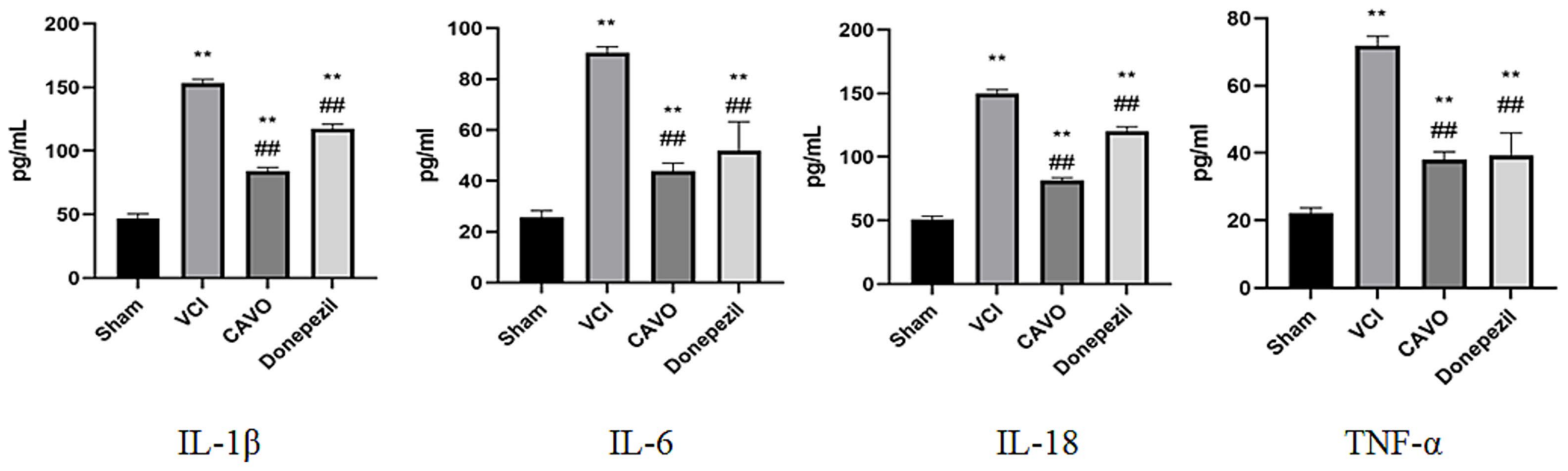
ELISA results for rat serum. Expression results of serum inflammatory factors in rats in the Sham, VCI, CAVO, and Donepezil groups. Compared with Sham group, *p < 0.05, **p < 0.01, and compared with VCI group, #*p* < 0.05, ##*p* < 0.01. * Indicates statistical difference from the Sham group and # indicates statistical difference from the VCI group.

Compared with the Sham group, the serum levels of interleukin 1β (IL-1β), interleukin 6 (IL-6), and tumor necrosis factor *α* (TNF-α) were significantly higher in the VCI group (*p* < 0.01). Compared with the VCI group, the serum levels of IL-1β, IL-6, IL-18, and TNF-α were significantly lower in the CAVO group (*p* < 0.01). Compared with the VCI group, serum levels of IL-1β, IL-6, IL-18 (150.10 ± 2.88 vs. 120.21 ± 3.60), and TNF-α were significantly lower (*p* < 0.01) in the Donepezil group, as shown in Figure 5.

#### 3.3.2 The effect of CAVO on the expression of proteins in the hippocampal tissue of VCI rats detected by western blot

**Figure 6:**
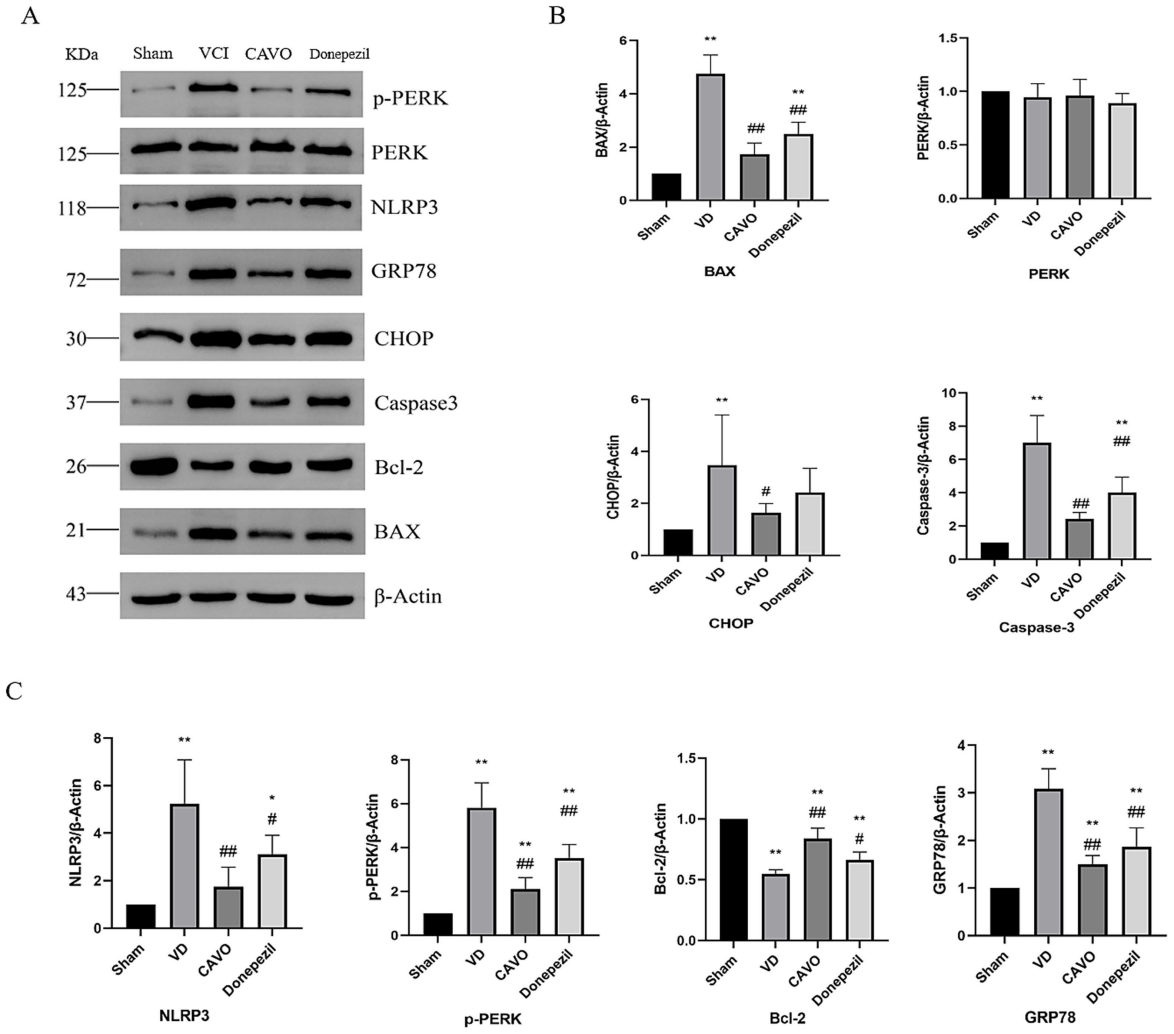
Results of Western blot experiments in the rat hippocampal region. **(A)** Protein blotting results of p-PERK, PERK, NLRP3, GRP78, CHOP, Caspase3, Bcl-2, BAX, and *β*-actin in rats in the Sham, VCI, CAVO, and Donepezil groups. **(B)** Relative expression of PERK, CHOP, Caspase3, and BAX in each group of rats. **(C)** Relative expression of p-PERK, Bcl-2, NLRP3, and GRP78 in each group of rats. Compared with Sham group, *p < 0.05, **p < 0.01 Compared with VCI group, #*p* < 0.05, ##*p* < 0.01.

Compared with the Sham group, CHOP, p-PERK, NLRP3, GRP78, BAX, and caspase3 protein expression was significantly higher (*p* < 0.01) and Bcl-2 protein expression was lower (*p* < 0.01) in the VCI model group. Compared with the VCI group, p-PERK, GRP78, NLRP3, BAX, and caspase3 protein expression was significantly lower (*p* < 0.01) in the CAVO group. CHOP protein expression was lower (*p* < 0.05), and Bcl-2 expression was significantly higher (*p* < 0.01) in the CAVO group compared with the VCI group. p-PERK, GRP78, BAX, and caspase3 protein expression was significantly decreased (*p* < 0.01), NLRP3 protein expression was decreased (*p* < 0.05), and Bcl-2 expression was elevated (*p* < 0.05) in the Donepezil group compared with the VCI group, as shown in Figure 6.

#### 3.3.3 PCR detection of mRNA expression in hippocampal tissue of VCI rats treated with CAVO

**Figure 7:**
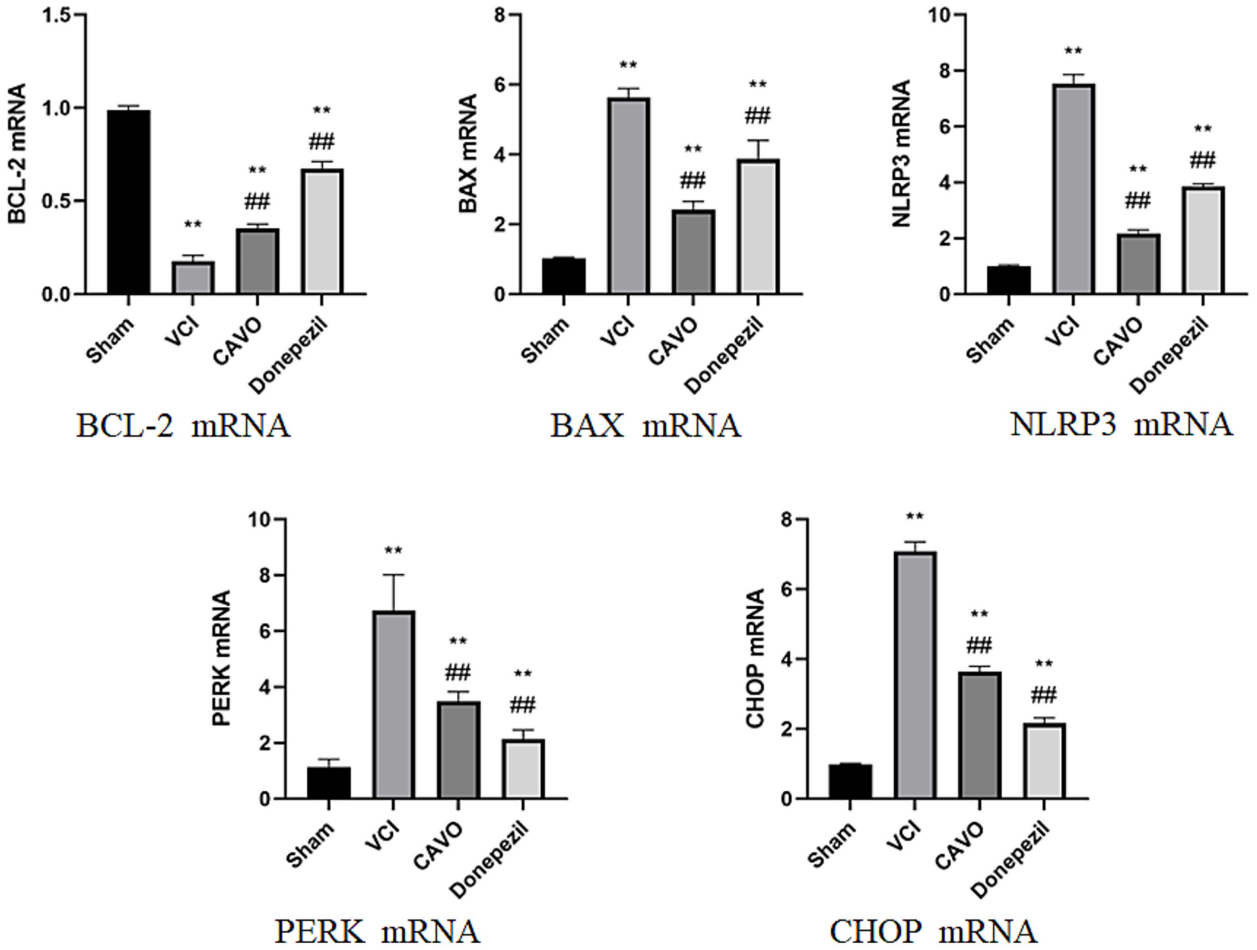
Results of PCR experiments in the hippocampus. PCR results of PERK, CHOP, NLRP3, Bcl-2, and BAX mRNA in rat cerebral cortex and hippocampus in rats in the Sham, VCI, CAVO, and Donepezil groups. Compared with the Sham group, *p < 0.05, **p < 0.01, compared with the VCI group, #*p* < 0.05, ##*p* < 0.01. * Indicates statistical difference from the Sham group and # indicates statistical difference from the VCI group.

Compared with the Sham group, CHOP, PERK, NLRP3 and BAX mRNA expression was significantly higher (*p* < 0.01) and Bcl-2 mRNA expression was lower (*p* < 0.01) in the VCI group. Compared with the VCI group, CHOP, PERK, NLRP3 and BAX mRNA expression was significantly lower (*p* < 0.01) and Bcl-2 mRNA expression was significantly higher (*p* < 0.01) in the CAVO and Donepezil (Figure 7).

### 3.4 Improvement of brain tissue morphology in VCI rats treated with CAVO

#### 3.4.1 HE staining

**Figure 8:**
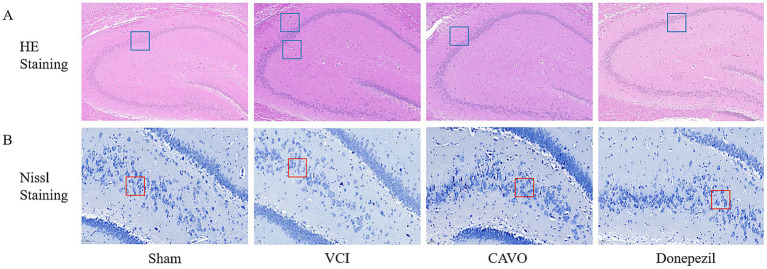
Results of pathological staining experiments in rat hippocampal area. Repair effects of CAVO treatment on brain cell damage in VD mice. **(A)** Nissl staining revealed morphological changes in neurons across different groups. Following administration, hippocampal cell morphology in the CAVO and Donepezil groups showed relative recovery, with increased hippocampal cell counts. This indicates that the treatment exerts a certain regulatory effect on hippocampal cells, whose outlines appeared clearer than those in the model group. **(B)** Histopathological assessment of hippocampal regions in all groups via HE staining. Compared to the model group, CAVO and Donepezil groups showed partial recovery of cell morphology and reduced numbers of atrophic cells. Scale bar = 50 μm, magnification 400×.

The hippocampal cell structure of rats in the Sham group was normal and well-defined, with round cells exhibiting clear boundaries and visible nucleoli. The cell structure of the hippocampal CA1 area was tightly and neatly arranged. Compared with the Sham group, the hippocampal cell structure of rats in the VCI group was denatured, with some cells solidified into pike and triangle shapes, as well as the absence of nucleoli and presence of vacuoles. The arrangement of neuronal cells in the hippocampal CA1 area was also loose, and the cytoplasmic staining was darker. Compared with the VCI group, the hippocampal cell structure was improved in the CAVO and Donepezil groups, with only a small amount of cellular solidification observed, and the cells in the CA1 area of the hippocampus were more neatly arranged (see Figure 8A).

#### 3.4.2 Nichols staining

The hippocampal CA1 area of Sham group rats has an abundance of nidus vesicles and a high number of cell layers, with the nidus vesicles arranged neatly and close together. Compared with the Sham group, the hippocampal CA1 area of the VCI rats exhibits a significant loss of nidus vesicles; the cells are scattered, the cell spacing is increased, and the number of nidus vesicles is evidently reduced. Compared with the VCI group, the hippocampal CA1 areas of rats in the CAVO and Donepezil groups exhibit a significant increase in nidus vesicles, the cell arrangement is tighter and more orderly, and the degree of cell damage is lower (see Figure 8B). The number of nitrosomes in the CA1 area of the hippocampus of the VCI group is obviously increased, the arrangement is tighter and more orderly, the number of hollow vesicles is increased, and the degree of cellular damage is lower (see Figure 8B).

#### 3.4.3 TEM observation of ultrastructural changes in the hippocampal region

**Figure 9:**
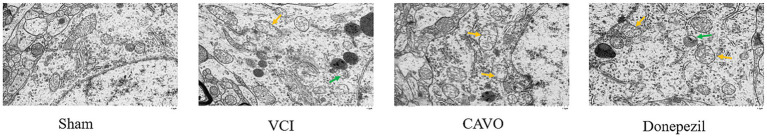
Transmission electron microscopy images of hippocampal organization. Transmission electron microscopy of mitochondria in the rat hippocampus (sham-operated group, VCI group, CAVO group, and Donepezil group). Scale bar = 1 μm, magnification ×12,000. Orange arrows indicate mildly swollen mitochondria; green arrows indicate autophagic vesicles.

The mitochondria in the Sham group were essentially normal, the inner and outer membranes were intact, the cristae were clear, and the electron density of the matrix was comparable to that of the cytoplasm. Compared with the Sham group, the mitochondria of the VCI group were swollen, the cristae were disorganized, and autophagic vesicles were visible. Compared with the VCI group, the nuclei of the CAVO group exhibited smooth nuclear membranes and slightly increased heterochromatin. The mitochondria were slightly swollen. Some mitochondria in the Donepezil group were essentially normal, inner and outer membranes were intact, cristae were well-defined, and the electron density of the matrix was comparable to that of the cytoplasm (see Figure 9).

#### 3.4.4 Immunofluorescence

**Figure 10:**
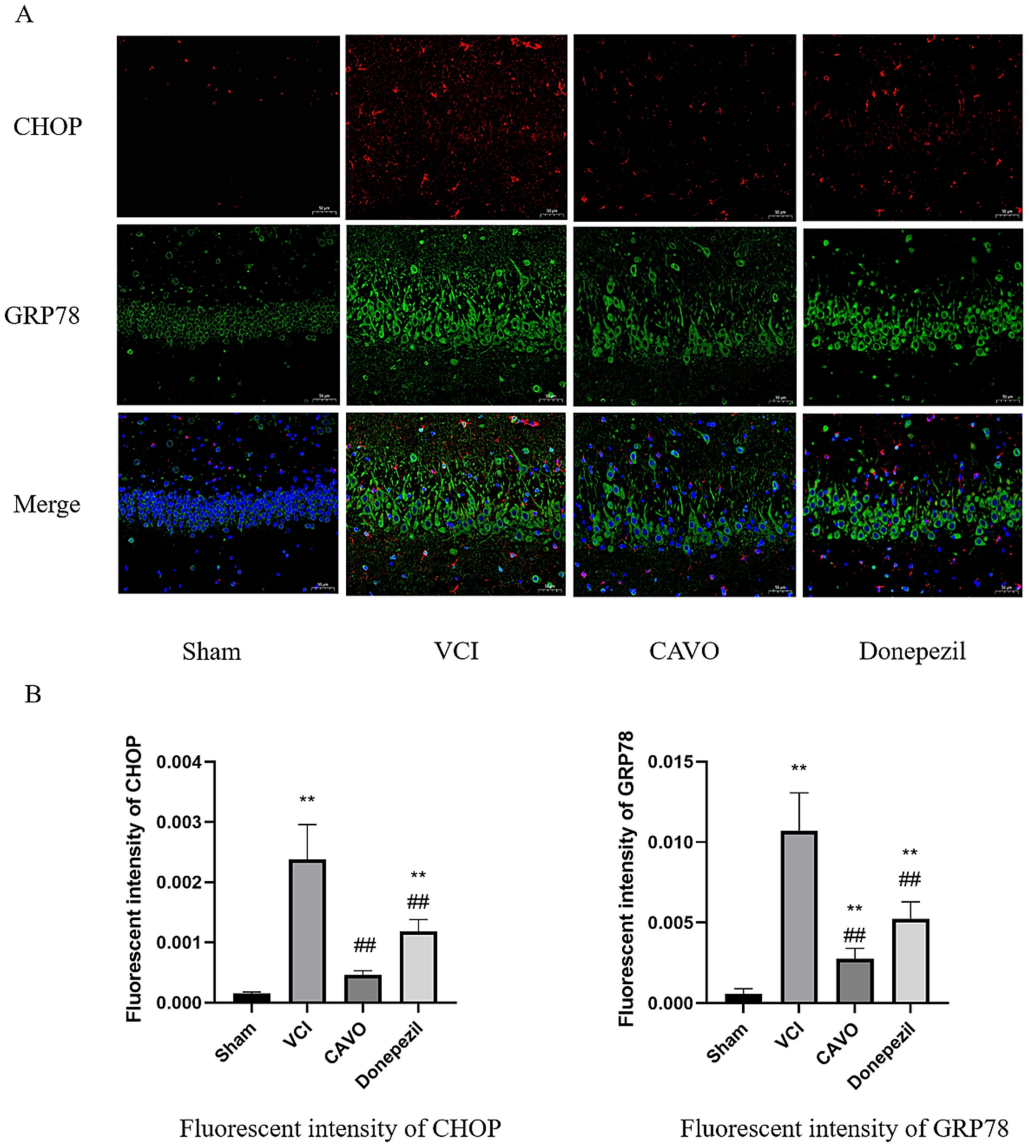
Immunofluorescence results of CHOP and GRP78 in rat brain tissue. **(A)** Immunofluorescence staining results for CHOP and GRP78 in the hippocampal tissues of rats in the Sham, VCI, CAVO, and Donepezil groups. Bars = 50 μm, magnification 400×. **(B)** Relative fluorescence intensity of CHOP and GRP78 in hippocampal tissues of each group. Values are expressed as mean ± SEM (*n* = 6). *p < 0.05, **p < 0.01 compared to Sham group, #*p* < 0.05, ##*p* < 0.01 compared to VCI group. * Indicates statistical difference from the Sham group and # indicates statistical difference from the VCI group.

**Figure 11:**
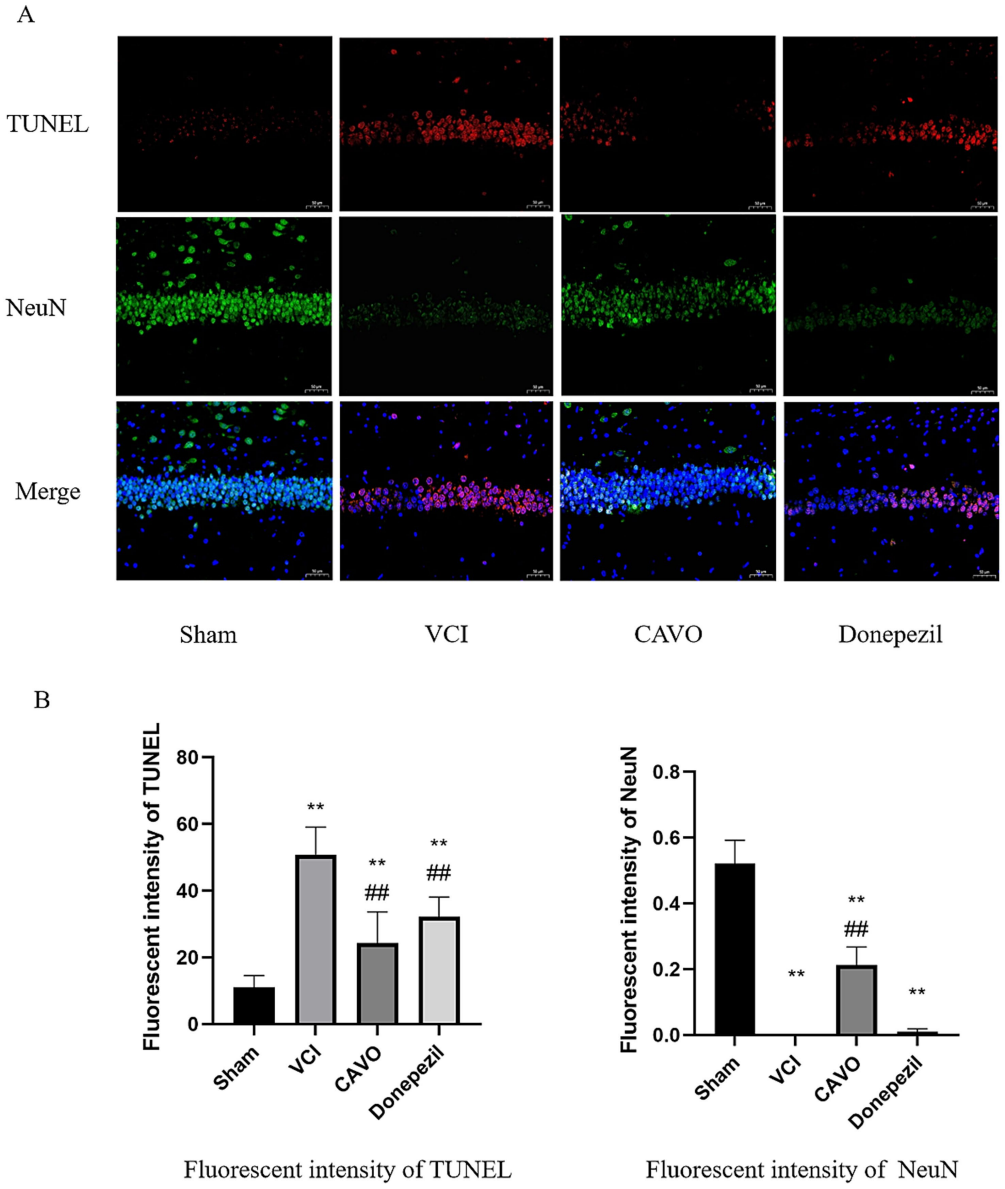
Immunofluorescence results of TUNEL and NeuN in rat brain tissue. **(A)** Immunofluorescence staining of TUNEL and NeuN in the hippocampal tissues of rats in the Sham, VCI, CAVO, and Donepezil groups. Bars = 50 μm, magnification 400×. **(B)** Relative fluorescence intensity of CHOP and GRP78 in hippocampal tissues of each group. Values are expressed as mean ± SEM (*n* = 6). *p < 0.05, **p < 0.01 compared to Sham group, #*p* < 0.05, ##*p* < 0.01 compared to VCI group. * Indicates statistical difference from the Sham group and # indicates statistical difference from the VCI group.

Immunofluorescence results revealed that the fluorescence intensity of CHOP, GRP78, and TUNEL was significantly higher (*p* < 0.01) and the fluorescence intensity of NeuN was significantly lower (*p* < 0.01) in the VCI group than in the Sham group. CHOP, GRP78, and TUNEL levels were significantly lower in the CAVO and Donepezil groups compared to the VCI group (*p* < 0.01), and NeuN fluorescence intensity was elevated (*p* < 0.01), as shown in Figures 10, 11.

## 4 Discussion

Our study demonstrated, for the first time, that CAVO modulates the ERS signaling pathway to improve cognitive function in a rat model of VCI. The VCI model was established by permanently ligating the common carotid arteries, verifying its success using the Morris water maze test, one of the most commonly used assessments for spatial learning and memory in neuroscience research. We found that the escape latency of rats in the VCI model group was significantly increased, indicating a progressive decline in spatial learning and memory abilities. These cognitive impairments are consistent with clinical symptoms of VCI, such as unresponsiveness, confusion, and memory loss (Niu et al., 2018). After administering CAVO by gavage for 14 days, the avoidance latency during the training period was significantly shortened, and the duration of stay in the target quadrant of the spatial exploration experiment was prolonged. This suggests that CAVO significantly improved the learning and memory abilities of VCI rats. Donepezil, currently the main drug used for treating VCI, also demonstrated therapeutic efficacy in the present study. In the subsequent neuroimaging and molecular mechanism studies, it was found that CAVO administered by gavage could reduce the inflammatory response, decrease apoptosis and necrosis, protect neurons in brain regions, enhance FC between brain regions, and play a role in improving cognitive function in VCI rats.

### 4.1 Alterations in rat brain function observed using fMRI

#### 4.1.1 Alterations in the function of memory-related brain regions in rats

The hippocampus is located in the medial temporal lobe of the brain and is part of the limbic system, which is closely related to emotion regulation, learning, and memory formation. Studies have confirmed that the hippocampus is atrophied in patients with VCI (Gao et al., 2023), which may be due to neuronal loss and reduced cerebral microvasculature. A 9.4T magnetic resonance study found that T2*, fractional anisotropy, and mean apparent diffusion coefficient values in the hippocampus of rats were decreased after bilateral common carotid artery occlusion, suggesting that insufficient blood supply to the hippocampal cells resulted in cellular edema. rs-fMRI uses ReHo to denote the temporal similarity of neuronal activity between a single voxel and its neighboring voxels. Higher ReHo values indicate that the temporal sequence of neuronal activity in neighboring brain regions is more consistent than the signal intensity, and lower ReHo values indicate that neuronal activity in neighboring brain regions is relatively disordered (Hung et al., 2024). The FC of brain networks can be evaluated using rs-fMRI, which measures interregional synchronization detected from BOLD fMRI sequences and is often used for early diagnosis of diseases in an attempt to establish a link between two spatial ROIs with the help of linear temporal correlations, which are inferred between brain regions based on correlations between neuronal activity parameters.

Memory is a complex process requiring coordinated activity across large-scale brain networks. The dorsal hippocampus (dHP), consisting of the dorsal regions of the dentate gyrus (DG), CA 3, and CA 1, plays an important role in situational memory (e.g., encoding, storage, and retrieval), and studies have demonstrated a close link between FC in the dHP and changes in memory (Han et al., 2024). Functions of the limbic cortex, a part of the subfrontal cortex, include controlling movement, managing verbal expression, and regulating emotion, and this region is primarily responsible for declarative long-term memory functions. Research has shown that ReHo values are elevated in the subfrontal cortex of patients with VCI, one of the key regions of the DMN (Yuan et al., 2021). This may be related to the DMN’s synergistic effects and the resting-state network in these areas during the pathogenesis of VCI, which aligns with our findings. It is hypothesized that the elevated ReHo values in the inferior frontal gyrus also play an important role in VCI pathogenesis.

#### 4.1.2 Functional changes in olfactory-related brain regions

The piriform cortex, the largest component of the primary olfactory cortex, is connected to multiple brain regions involved in functions such as olfaction, learning and memory, sleep, and is also associated with neurological conditions such as epilepsy. Kumar et al. (2021) demonstrated that the acquisition and formation of cognitive memories are largely dependent on the neural connections between the olfactory bulb and the piriform cortex. In patients with cognitive impairment, FC between the posterior cingulate cortex and anterior cingulate cortex is reduced, and our results are consistent with previous findings (Ibrahim et al., 2021). Reduced connectivity between the cingulate cortex and the piriform cortex is frequently observed in olfactory disorders and may affect foraging behavior in rats.

Studies have shown that many age-related dementias, such as Alzheimer’s disease, vascular dementia, Parkinson’s disease, and frontotemporal lobe dementia, often involve olfactory dysfunction (Alves et al., 2014), which may be a promising direction for future research on dementia-related diseases.

#### 4.1.3 Functional changes in auditory-related brain regions

The medial geniculate is a small structure located below the occipital part of the thalamus and is connected to the inferior colliculus of the midbrain via the inferior colliculus arm. It is a subcortical auditory center together with the inferior colliculus. If a CNS lesion affects the medial geniculate, tinnitus, hearing impairment, and other changes can occur. In patients with semantic dementia, there is a decrease in gray matter in the medial geniculate body (Mahoney et al., 2011).

Hearing loss (HL) and cognitive dysfunction are prevalent. Cross-sectional and longitudinal studies have shown that peripheral HL is associated with cognitive dysfunction, including AD and VCI. One study examined PET biomarkers using PET, MRI, and audiometric testing, finding that peripheral HL may be associated with *β*-amyloid deposition (Jung et al., 2021). Mendelian randomization studies have even demonstrated a significant association between hearing and vision impairment and increased risk of dementia; therefore, standardized hearing and vision assessment and intervention should be emphasized in dementia prevention strategies (Jiang et al., 2024). *The Lancet* published its 2024 report on dementia prevention, intervention, and care, which identified hearing loss as one of the 14 risk factors for dementia and suggested that attention should be paid to early hearing impairment in patients with dementia (Livingston et al., 2024).

Our study found that brain regions related to olfaction (e.g., piriform cortex), hearing (e.g., medial geniculate), and spatial cognition (e.g., dorsal hippocampus, and prelimbic/infralimbic) were abnormal in various respects, including function and FC. If such abnormalities are detected early, there is the potential to mitigate dementia symptoms and progression during its early stages.

Elevated levels of inflammatory markers, including IL-1β and IL-6, have been found in brain tissue and blood samples from VCI patients (Ng et al., 2018). The 2-VO surgical model we used leads to neuronal loss and increased apoptotic cells in the hippocampus, as well as impaired spatial memory (Khoshnam et al., 2018). Our experiments found that the expression of apoptosis-related proteins was elevated in rats in the VCI group, suggesting that inflammatory responses can affect hippocampal function, which can, in turn, lead to cognitive deficits. CAVO administered by gavage significantly improved IL-1β, IL-6, and TNF-α serum levels and attenuated the expression of NOD-like receptor protein 3 (NLRP3) inflammatory vesicle proteins and mRNAs in the hippocampal tissues of the VCI rats. This suggests that CAVO can inhibit inflammatory responses in rats *in vivo*, corroborating the results of previous studies (Lee et al., 2022; Chen et al., 2024). The underlying mechanism of this improvement may be CAVO’s ability to inhibit the production of TNF-α, IL-1β, and nitric oxide (NO) in microglia, thereby exhibiting strong anti-inflammatory and antioxidant effects.

NLRP3 catalyzes the release of IL-1β and IL-6 by activating cysteine protein hydrolase 1 (caspase-1), an activated caspase-1. In addition, NLRP3 inflammatory vesicles, a complex of NLRP3, and apoptosis-associated speck (ASC)-like proteins, which induce programmed cell death under stressful and inflammatory pathological conditions, are important pattern recognition receptors in the cell cytoplasm and play a very important regulatory role in immune and inflammatory responses (Xu et al., 2024). In the VCI rats, bilateral common carotid artery surgery induced the activation of inflammasome components (NLRP3 and caspase 1), the release of their downstream products (IL-1β and IL-6), and the activation of pro-apoptotic cell death, which was confirmed by our findings (Cordaro et al., 2022). Studies have shown that NLRP3, pro-apoptotic Bcl-2-related X protein (BAX), and anti-apoptotic protein B-cell lymphoma-2 gene (Bcl-2) are closely associated with the development of dementia (Dai et al., 2020). Our study found significantly elevated BAX and caspase-3 protein expression and significantly reduced Bcl-2 protein and mRNA expression in VCI rats after gavage administration of CAVO compared with the other groups. The HE staining results showed that the neuronal cells in the CA1 area of the hippocampus of rats in the VCI group were loosely arranged, and the cytoplasmic staining became darker, suggesting that apoptosis had occurred. This was improved after CAVO treatment, showing that CAVO treatment prevented cell apoptosis.

The endoplasmic reticulum (ER) is a key organelle that regulates protein folding, transport, and post-transcriptional modifications, and is involved in the synthesis and metabolism of lipids and steroids. When various internal and external environments stimulate the ER, causing unfolded or misfolded proteins to accumulate in the lumen, a pathological state called ERS develops (Wang and Kaufman, 2016). Dysregulation of redox homeostasis, inflammation, hypoxia, ischemia–reperfusion injury, and many other factors can interfere with ER homeostasis, and ER function imbalance causes the unfolded protein response (UPR), which activates inflammatory and apoptotic signaling pathways, leading to cellular dysfunction. The UPR is a major mechanism mediating ERS, mediated by three transmembrane proteins—activating transcription factor 6 (ATF6), the double-stranded RNA-dependent protein kinase R-like endoplasmic reticulum kinase (PERK), and inositol-requiring enzyme 1 (IRE1). In the early ERS phase, the UPR first indirectly inhibits protein synthesis and RNA transcription by phosphorylating PERK (p-PERK), mediated by the phosphorylation of eukaryotic initiation factor 2α (eIF2α) to reduce ERS loading. After ATF6 is released, it translocates to the Golgi and cleaves into ATF6 fragments, which act as active transcription factors to promote glucose-regulated protein 78 (GRP78), glucose-regulated protein 94 (GRP94), glucose-regulated protein 95 (GRP96), and other folding-promoting genes to correct ERS imbalance. When prolonged ERS impairs ER function, PERK, ATF6, and IRE-1α initiate apoptotic signaling pathways mediated by ERS to induce apoptosis and promote the development of VCI (Liu et al., 2022). Caspase, CHOP, and JNK synergistically initiate apoptosis-related signaling, inhibit Bcl-2, and activate BAX expression. ERS regulates the expression and activation of NLRP 3 through different mechanisms, such as oxidative stress and CHOP-dependent NF-κB activation, which induces the production of inflammatory factors, such as NLRP3 and interleukin (IL). In different experimental models of VCI, chronic ER dysfunction is highly correlated with memory and cognitive performance (Lourenco et al., 2013). In the present study, protein and mRNA expression of CHOP, PERK, and GRP78 in the ERS pathway were elevated in the hippocampal tissues of VCI rats after inadequate cerebral perfusion. Apoptotic factors caspase-3 and BAX were also up-regulated in hippocampal tissues in the VCI group. These results suggest that ERS and apoptosis play important roles in the pathogenesis of VCI in rats, and that persistent ERS can lead to impaired cognitive function by a mechanism related to the activation of the ERS-induced apoptotic pathway (Lin et al., 2019). The cognitive deterioration of the VCI rats, as evidenced by our water maze results, could also confirm this conclusion. The protein and mRNA of inflammatory factor (NLRP3) and apoptotic factor (BAX, Bcl-2, Caspaes3) in the ERS pathway were improved after CAVO treatment.

## 5 Limitations and prospects

In this study, we used rs-fMRI to explore the mechanism of CAVO in treating VCI, calculating ReHo value, gradient value, and FC as measures of cognitive function. However, due to the relatively small sample size, we were unable to perform multiple comparison corrections, an acknowledged limitation common to imaging studies using animal models. In future research, the use of pathway-related agonists and inhibitors may help to further explore the specific mechanism of CAVO in the signaling pathway.

## Data Availability

The datasets presented in this study can be found in online repositories. The names of the repository/repositories and accession number(s) can be found in the article/Supplementary material.

## Footnotes

^1^ http://stnava.github.io/ANTs/
