# Supplementary figures and images for: Neuroimaging and molecular mechanism of action of Cang-ai volatile oil in the treatment of vascular cognitive impairment
Source: Front Neurosci. 2025 Nov 3;19:1688649. doi: 10.3389/fnins.2025.1688649 (PMC12620445; doi:10.3389/fnins.2025.1688649)

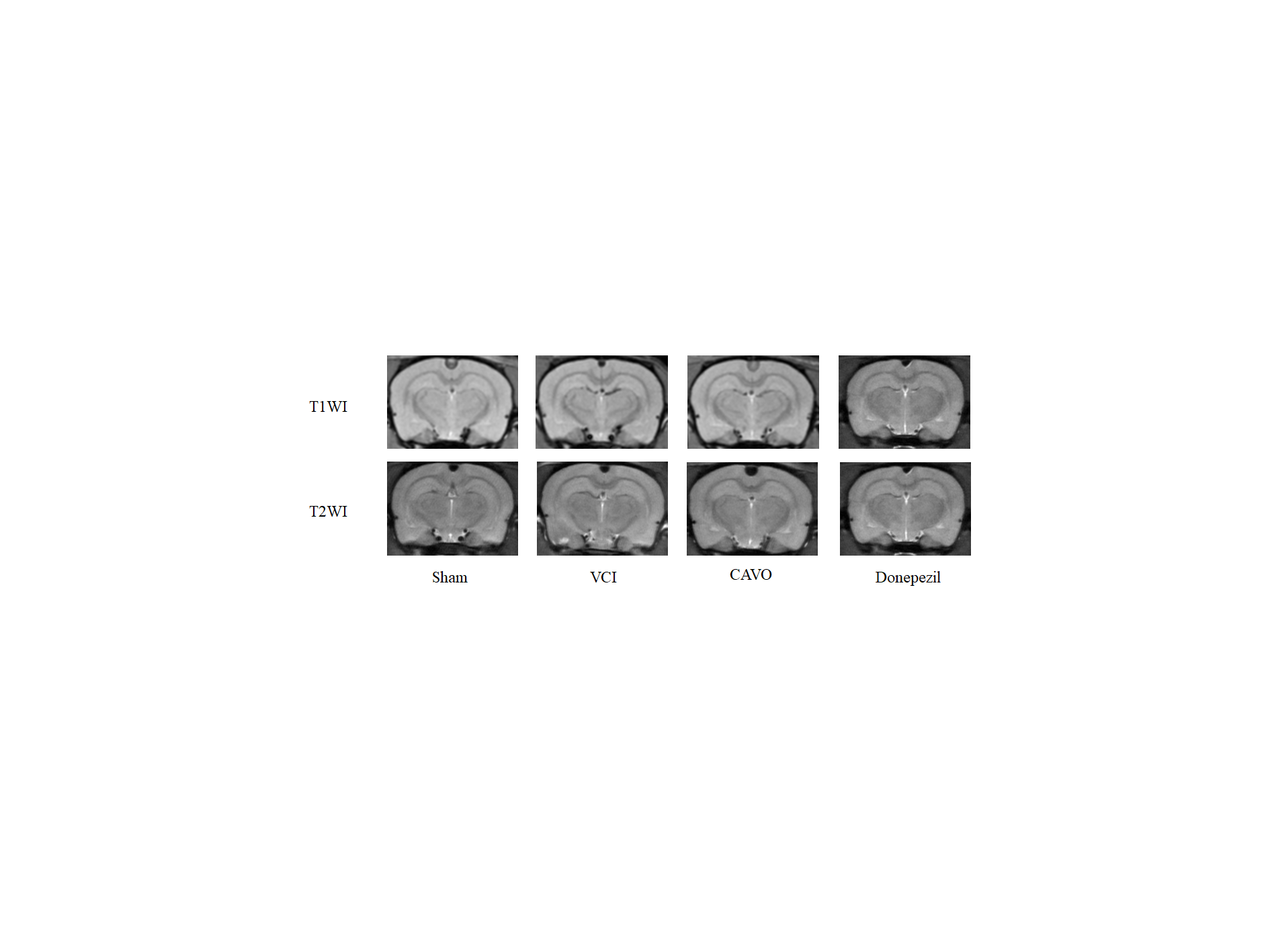

Supplement: Supplementary file 1 [file Image_1.tif]
